# Parapharyngeal space emphysema by temporal bone fracture resulting from mandibular trauma

**DOI:** 10.5935/1808-8694.20130095

**Published:** 2015-10-08

**Authors:** José Pedro Beira de Matos, Pedro Jorge Oliveira, Manuela Campos Ferreira, Artur Condé

**Affiliations:** a Resident physician (Resident at the Vila Nova de Gaia Hospital Center/Espinho - Portugal).; b Hospital assistant at the Vila Nova de Gaia Hospital Center/Espinho - Portugal).; c Service Director (Service Director - Vila Nova de Gaia Hospital Center/Espinho - Portugal). Vila Nova de Gaia Hospital Center/Espinho - Portugal.

**Keywords:** emphysema, external acoustic meatus, mandible trauma, temporal bone

## INTRODUCTION

The parapharyngeal space has been studied throughout the years, with different interpretations concerning its limits^1^. One of the important limits to bear in mind when dealing with trauma is its lateral wall, formed by the mandibular ramus. The mandible is the only mobile bone in our faces and it is frequently affected by trauma because of its projection on the inferior third of the face^2^. The temporomandibular joint (TMJ) is also the site of direct or indirect trauma by means of complications arising from mandibular trauma^3^. There are rare cases of external ear meatus fracture by mandible trauma^4^. This clinical case reports an external acoustic meatus fracture, causing an emphysema in the ipsilateral parapharyngeal space consequent to the mandible trauma.

## CASE PRESENTATION

A 54-year-old male patient came to the Urgency Ward because of a mandible trauma caused by an accidental fall. He reported mild pain in the TMJ region. He reported no hearing loss and no other relevant symptom. Upon objective examination, he had a mild edema on his left TMJ region, with otorrhagia in the ipsilateral external acoustic meatus. His mandible mobility was normal, with mild pain upon TMJ palpation. The otoscopic exam revealed a depression on the anterior wall of the external acoustic meatus, on its bony portion in the middle third, with no other lesions. There was no evidence of other types of head and neck trauma.

The CT scan showed an emphysema in the parapharyngeal space and a fracture on the external acoustic meatus anterior wall. There was no other type of lesion on the mandible or temporal bone fracture.

**Figure 1:**
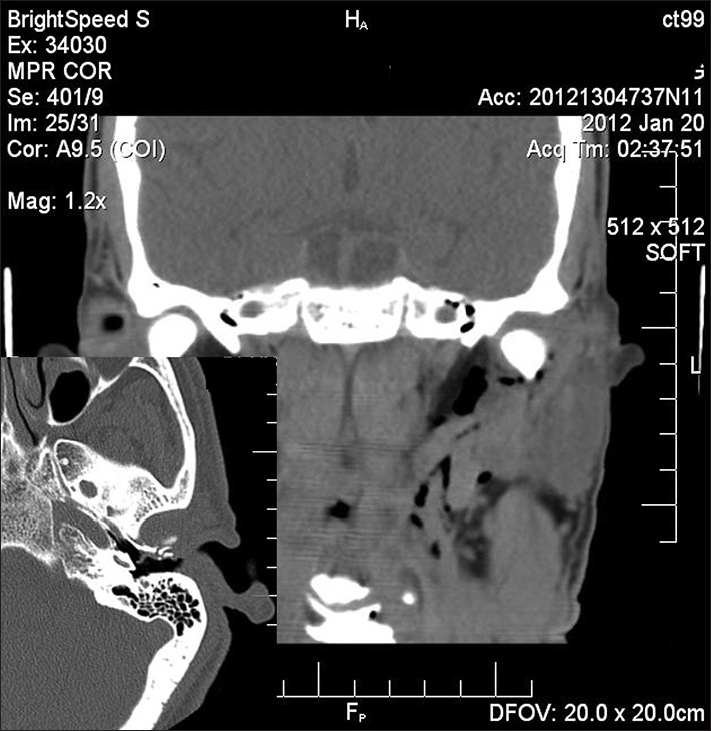
Coronal and axial CT images. External auditory canal anterior wall fracture and consequent emphysema of the ipsilateral parapharyngeal space.

We decided to observe the patient only and he was followed up in the office. His emphysema subsided within two weeks. He had no more complications along his clinical follow up (Figure 1).

## DISCUSSION

Mandible trauma is a frequent lesion in frontal facial trauma and a fracture is a possible consequence, happening more frequently in the mandible body and condyle^5^. Because of its topography, the mandible may indirectly cause another type of trauma, without suffering a fracture itself. This is the case of temporal bone fracture, namely of the external acoustic meatus, more specifically on its anterior wall^6^.

This case reports this less common knowledge and the rare form a complication of the same fracture, with the emphysema ensuing in the ipsilateral parapharyngeal space. This one formed because of the very fragility of its lateral border, formed by the mandible ramus, and the consequent air penetration.

It is always necessary to carefully interpret image studies, and when one detects an emphysema in the neck, one must always look for the lesion that caused it^6^.

In this particular case, such association was suspected because of the blood in the ear and the objective exam, and it was confirmed by the image exam.

## FINAL REMARKS

Mandible trauma may be translated in many ways. The parapharyngeal emphysema as a complication of external acoustic meatus fracture, must be considered in very specific cases of mandible trauma. The participation of an otorhinolaryngologist was paramount for the diagnosis, and this fact stresses the importance of multidisciplinary care for trauma in general and facial traumas must really have it.
